# Protein and Lipid Digestibility of Pasture-Raised and Grain-Finished Beef: An In Vitro Comparison

**DOI:** 10.3390/foods12061239

**Published:** 2023-03-14

**Authors:** Lovedeep Kaur, Amrutha Elamurugan, Feng Ming Chian, Xianqian Zhu, Mike Boland

**Affiliations:** 1 School of Food and Advanced Technology, Massey University, Palmerston North 4442, New Zealand; amru.5297@gmail.com (A.E.); f.chian@massey.ac.nz (F.M.C.); 2 Riddet Institute, Massey University, Palmerston North 4442, New Zealand; x.q.zhu@massey.ac.nz (X.Z.); m.boland@massey.ac.nz (M.B.)

**Keywords:** pasture-fed, grain-finished, meat, in vitro digestion, protein nutrition, essential fatty acid, beef, tenderloin, striploin, protein digestion, lipid digestion, grain-fed, grass-fed

## Abstract

This study compared the digestibility of protein and fat components of pasture-raised and grain-finished beef using an in vitro oral-gastro-small intestinal digestion model. Two commonly consumed beef cuts, tenderloin (*Psoas major*) and striploin (*Longissimus dorsi*) were selected for this study. There were no substantial differences between the pasture-raised and grain-finished cuts of meat in terms of protein digestibility, as shown by the protein and peptide breakdown (observed through SDS-PAGE) and the degree of hydrolysis as measured by free amino nitrogen. Tenderloin, however, showed significantly (*p* < 0.05) higher overall protein digestibility than striploin. Both striploin and tenderloin digests from pasture-raised beef released significantly (*p* < 0.05) higher total amounts of free long-chain *n*−3 PUFAs and lower amounts of many free saturated fatty acids, notably palmitic and myristic acids, than those from grain-finished animals. The results suggest greater health benefits from consuming pasture-raised beef, particularly tenderloin.

## 1. Introduction

Meat is recognised nutritionally as a good source of protein and iron. It is also a source of important long-chain polyunsaturated fatty acids. However, any nutritional value depends on bioavailability. Meat digestion is a complex phenomenon. Generally, red meat contains about 70–75% water, 5–20% fat (depending on the cut), 16–23% protein, and 3.5–5% non-protein substances and inorganic compounds [1,2]. Years of research have shown the importance of meat as a protein source in the human diet. Although meat protein is known to be highly digestible, it has been argued that residual undigested meat protein in the colon can change the metabolism (and eventually the population structure) of the microbiome, which may lead to adverse health outcomes [3]. Thus, meat with higher digestibility will be seen as healthier. In addition to this, it has been argued that higher rates of release of amino acids during the digestion of meat will cause desirable anabolic effects in muscle, leading to maintenance or gain of muscle mass. This is particularly important for the elderly in managing sarcopenia (muscle wasting) and for athletes or fitness seekers who want to strengthen muscles [4].

The presence of saturated fatty acids and the fat content in meat has been related to health complications in some epidemiological studies conducted in western countries, although more research is being conducted to confirm this [2]. Contrary to popular perception, the fat content in many retail beef cuts has been declining in recent years, due to more effective fat trimming, the production of leaner cattle breeds, and enhanced animal husbandry practices. Hence, it has become crucial to communicate these changes to consumers [1]. The incorporation and moderate consumption of lean red meat in the diet have been shown to positively influence nutrient uptake and overall long-term health [5]. However, the effects of the breed, sex, and diet of animals, especially of pasture- and grain-based diets, need to be studied and reported to understand the breakdown of meat components and their implications for the maintenance of good health. There are studies in the literature that compare the composition, colour, and texture of pasture-raised and grain-finished beef [6,7,8,9,10]. These studies have recognized the differences in the fatty acid composition of the meat, but as yet there is little knowledge in the literature about how red meat is digested in the human digestive tract and how pasture-raised may differ from grain-finished meat, particularly with respect to the kinetics of protein digestion and the digestion and release of lipids, including the fatty acids that are important for health. We have no information yet about the digestion of meat lipids, but it is clear that pasture-raised meat contains significantly enhanced amounts of some of the nutritionally important polyunsaturated fatty acids [9,11,12]. Because the structure of the meat is based around protein, higher rates of digestion of protein are likely to influence the release of fat from within the structures, potentially affecting its digestion and bioaccessibility.

The individual amino acid concentrations of meat can potentially be altered by using genetic or feeding strategies, but further research is needed to evaluate their potential to alter the total protein content of meat [13]. Because cattle are ruminants, i.e., foregut fermenters, bioaccessible nutrients comprise the products of fermentation and the remaining undigested or partially digested components of their forage. The composition of the fat in an animal is largely affected by what it has been eating and the results of rumen fermentation. Most of the fat in meat comprises triglycerides. The fatty acid composition of the triglycerides dictates their physical characteristics and the health benefits of consuming the fat. Cattle obtain the precursors for fat synthesis from their diet. Fat that is taken in, for example from plant origin, is broken down to fatty acids in the rumen, and a degree of saturation (removal of double bonds) and breakdown of the fat occurs in the rumen. Anaerobic metabolism in the rumen (notably of carbohydrates) leads to the production of short-chain fatty acids, mostly acetic (C2) and propionic (C3) acids. Starch that is taken in (mostly in grain-fed animals) is metabolized into short-chain fatty acids. The animal can synthesize longer-chain fatty acids from C2, but only up to C16; thus, starch cannot be a precursor for longer-chain fatty acids [14]. Longer-chain fatty acids must be derived from the food source, although they can be modified by the animal through elongation and desaturation reactions. It can be expected that differences in the composition of intramuscular fat will have an effect on muscle structure and hence affect the digestion of meat.

The present study was therefore designed to understand the protein and fat digestion of New Zealand beef (striploin and tenderloin) from pasture-raised and grain-finished animals using an internationally recognised in vitro model for human digestion. This study was based around commercially produced pasture-fed and grain-finished beef, so could not be strictly controlled for individual animal diets, but it does reflect normal farming conditions in New Zealand and thus typical New Zealand beef. All the samples were cooked using standard procedures before the digestions and analyses.

## 2. Materials and Methods

### 2.1. Materials

All the animals used for the meat in this trial were specifically selected from the wider population as a common type. They were selected to be prime steers of the Aberdeen Angus breed, with a carcass weight of 324–343 kg.

Pasture-fed means that the animals have been raised under normal New Zealand farming conditions with year-round access to grass (e.g., hay, silage, Lucerne, feed crops, or other grazed or conserved forages) and other supplementary feeds [15]. In late 2019, five pasture-raised Aberdeen Angus steers, aged between 18 and 36 months, that had been grazed free range on pastures of predominantly perennial ryegrass and white clover were sourced from three farms that supply Silver Fern Farms Ltd. They were slaughtered on the same day in a commercial beef abattoir (Silver Fern Farms Whakatu plant, Hastings, New Zealand) according to humane standard operating procedures.

Five additional grain-finished Aberdeen Angus steers, aged 26–28 months, were sourced from the Canterbury feedlot of Five Star Beef where they were finished at an average of 122 days on a ration of maize silage, barley wheat, and straw, producing an average daily gain of 1.4 kg. All animals were pasture-fed as per the definition of pasture-fed by the Animal Status Declaration [15] until the beginning of the grain finishing for the grain-finished animals. They were similarly slaughtered (ANZCO Foods Canterbury Ashburton plant site, New Zealand).

The carcasses of the pasture-raised and the grain-finished beef were chilled for 24 h post-slaughter, and their pH was measured in the abattoir. Then the selected beef cuts (striploin—*Longissimus dorsi*, and tenderloin—*Psoas major*) were collected from the left side of the carcasses, vacuum packed and aged for 21 days at −1.5 °C (consistent with normal cold ageing, [16]) before sub-dividing into 1-inch-thick steaks. The steaks were individually vacuum-packed and stored at −20 °C until they were used for the digestion experiments. More details on the carcasses are given in Table S1.

The striploin and tenderloin steaks were chosen, as they have relatively less connective tissue and hence are the most tender cuts with good marbling and strong beef flavour. The fat on the striploin is easier to trim and there are no large pockets of fat, which favours a faster cooking time and makes it easier to cut. Tenderloin steaks are usually sold defatted with the chain muscle removed.

All chemicals and reagents used in this study were analytical grade.

### 2.2. Methods

#### 2.2.1. Cooking of Striploin and Tenderloin Steaks

The meat was cooked using the method of Purchas and Wilkinson [17]. The selected frozen steak was submerged in water at room temperature for 1 min to thaw the surface for easier cutting of the frozen meat. The meat was then cut into approx. 50 g steaks, vacuum packaged and stored at 4 °C to thaw for 16–20 h. The thawed steak was pat-dried, weighed and cooked in an electric skillet (ZIP non-stick electric skillet 26 cm dia.) with a surface temperature of 220–230 °C. The meat was cooked for 2.5 min on each side initially and additionally for 1 min on each side until it reached an internal temperature of 67 ± 2 °C. The cooked meat was rested for 10 min, pat dried and weighed to estimate cook loss. The subcutaneous fat layer of the striploin steak was removed after cooking to reflect normal use when eating the steak.

The cooked meat samples were used for analysing physicochemical parameters and in vitro protein and fat digestion as explained in Section 2.2.2 and Section 2.2.3.

#### 2.2.2. Physicochemical Analysis of Meat

##### Cook Loss Measurements

The cooking loss was estimated as the difference in weight of the samples before and after cooking expressed as a percentage of the weight before cooking [18]:

$$cook\;loss\;\left(\%\right)=\frac{\left(sample\;wt.before\;cooking-sample\;wt.after\;cooking\right)}{sample\;wt.before\;cooking}\times 100$$

##### Moisture Analysis and pH

The pH of the cooked meat samples was measured using the protocol mentioned by Zhu [19].

Moisture content in cooked samples was analyzed using the AOAC 950.46 method [20]. A conventional electric oven was set to 105 °C with consistent airflow and heat distribution. Two to three grams of cooked samples were placed in individual aluminium dishes and weighed. They were oven-dried for 16 h, cooled in a desiccator and weighed. The percentage loss in weight was reported as moisture content.

##### Crude Protein (%) and Fat (%) Analysis

The protein (%) in cooked samples was analysed using the Kjeldahl method [21]. The test was performed in triplicate for each of *n* = 5 pasture-raised samples and *n* = 5 grain-finished samples.

The fat content of the samples was determined using the Mojonnier (acid) method (Flour, Baked, extruded products) [22].

#### 2.2.3. In Vitro Digestion Experiments

The in vitro digestion of the meat samples was conducted as described by Chian et al. [23] using the INFOGEST method [24] with some modifications. Three replicates were performed per sample. For each replicate, two separate in vitro digestion experiments were performed in individual double-jacketed glass reactors at 37 °C with chosen sampling times.

For each digestion 8 g of meat was ground using a mortar and pestle for a minute, followed by incubating the samples with 8 mL of simulated salivary fluid containing 1.25 × 10^−6^ katal/mL bolus of α-amylase (10025, Sigma Aldrich, Saint Louis, MO, USA) at pH 7 ± 0.1 for 2 min to simulate chewing and oral digestion. Subsequent simulated gastric digestion was initiated by the addition of 32 mL of simulated gastric juice containing 1.33 × 10^−7^ katal/mg meat protein of porcine pepsin (P7000, Sigma Aldrich, Saint Louis, MO, USA) pH 3 ± 0.1. Three glass balls (3–5 mm dia.) were added to mimic meat maceration in the stomach. Sampling was conducted after 0 and 60 min of gastric digestion in the first reactor. Pepstatin A (12 μL, ab141416, Abcam, Cambridge, UK) (0.5 mg/mL in methanol) was immediately added after sampling to every 1 mL of the gastric digests.

In a second reactor, in vitro small intestinal digestion was initiated after an hour of gastric digestion as described above. Small intestinal digestion was commenced by adding 48 mL of simulated small intestinal fluid and pancreatin (P1750, Sigma Aldrich, Saint Louis, MO, USA) at 1:100 pancreatin to meat protein ratio into the gastric digests. The pH of the digest was adjusted and maintained at pH 7 ± 0.1 with constant mixing. The sampling was done after 10, 60, and 120 min of small-intestinal digestion.

An aliquot of 0.45 mL of SIGMAFAST™ protease inhibitor cocktail solution (S8820, Sigma Aldrich, Saint Louis, MO, USA) (one tablet in 50 mL Milli-Q water) was mixed with every mL of small-intestinal digest after sampling to inactivate the digestive enzymes.

The mixtures of digests and enzyme inhibitors were homogenized for 30 s using a homogenizer with a 5 mm diameter disperser element at setting 3 (T10 basic ULTRA-TURRAX^®^ IKA Werke GmbH & Co. KG, Staufen im Breisgau, Germany). Finally, the digests were immersed in an ice bath and then stored at −20 °C until further analysis.

#### 2.2.4. Assessment of Protein Digestibility

##### Ninhydrin Analysis of Digests

Digests were thawed and centrifuged at 14,100× *g* for 3 min (Eppendorf Mini spin plus, Hamburg, Germany) at room temperature. The supernatant was filtered through a 0.45 μm PVDF filter (Millex^®^, Duluth, MN, USA) before analyzing for ninhydrin reactive amino N [25] using ninhydrin reagent (N7285, Sigma Aldrich, Saint Louis, MO, USA). A standard curve was prepared using a stock solution of 50 μM glycine in 0.05% glacial acetic acid.

##### Tricine SDS-PAGE of Digests

The homogenized digests were examined for the breakdown of specific proteins using reduced-Tricine-SDS-polyacrylamide gel electrophoresis (SDS-PAGE) as described by Chian et al. [23]. The digests were mixed with tricine sample buffer (Bio-Rad Laboratories, Hercules, CA, USA), then 25 µL of each sample was loaded into individual wells (16.5% gradient Tricine gels, Criterion^TM^ Gel, Bio-Rad Laboratories, Hercules, CA, USA) at a protein concentration of 1 mg/mL. Gels were run using a CriterionTM cell (Bio-Rad Laboratories, Hercules, CA, USA) at 125 V and then stained with Bio-safe Coomassie blue stain (Bio-Rad Laboratories, Hercules, CA, USA). The gel was scanned with a Gel Doc XR + Gel Documentation System (Bio-Rad Laboratories, Hercules, CA, USA), followed by analysis using Image Lab^TM^ software (version 6.1.0, Bio-Rad Laboratories, Hercules, CA, USA).

#### 2.2.5. Free Fatty Acid Analysis of Digests

The free fatty acids were analyzed in the meat digests at the end of digestion (180 min) using the method of Zhu et al. [26]. The contribution of lipolysis in the gastric phase was not considered because lipolysis in the stomach has been estimated to be only about 10% of total lipolysis, and gastric lipase contributes nearly as much lipolysis in the intestinal phase as it does in the stomach [27]. Mackie and Rigby [28] also reported that gastric lipolysis is limited in general due to the saturation of relatively low pH lipid interfaces. The unavailability of suitable and affordable lipase is also one of the reasons why gastric lipase is not normally used during in vitro digestion experiments and therefore a reason for not studying gastric lipolysis of meat in this study.

##### Methylation of Total Fatty Acids (TFA)

The total amount of individual fatty acids (TFA), including free fatty acids (FFA) and esterified fatty acids in monoglyceride, diglyceride, and triglyceride forms, were analyzed in the ground freeze-dried control cooked meat samples and their digests using the method described by Zhu et al. [26].

A freeze-dried meat sample or digest (100 mg) was weighed into a glass tube. An internal standard of methyl tricosanoate was prepared in heptane (1 mg/mL). One millilitre was added to the glass tubes. Potassium hydroxide (0.7 mL of 10 M) and 5.3 mL of methanol were then added and the contents vortexed. The tubes were incubated for 90 min at 55 °C and vortexed individually for 5 s every 20 min. The tubes were then cooled to room temperature and 0.58 mL of 12 M H_2_SO_4_ was added to form a precipitate of K_2_SO_4_. The tubes were then reincubated and similarly vortexed for another 90 min. The tubes were cooled, and the fatty acid methyl esters (FAMEs) were extracted as follows: 3 mL of heptane was added, the tubes vortexed and then centrifuged at 3500 rpm for 10 min. The upper layer was collected in a new glass tube containing a 1 mm bed of anhydrous Na_2_SO_4_. Finally, the heptane layers containing methylated total fatty acids were collected in 2 mL GC vials and stored at −20 °C until GC analysis.

The procedure was done in triplicate for each sample.

##### Methylation of Ester Forms of Fatty Acids

Ester forms of the fatty acids (EFA) in the cooked meat samples and their digests that include monoglyceride, diglyceride, and triglyceride forms were determined by the sodium methoxide transesterification method as described by Zhu et al. [26].

Ground freeze-dried (100 mg) samples of control meat or its digest were weighed in glass tubes and 1 mL of the internal standard was added. Next 1 mL of sodium methoxide solution was added, the contents were vortexed and then incubated for 60 min at 55 °C. The tubes were cooled to room temperature and 0.1 mL of glacial acetic acid, 5 mL of saturated NaCl solution, and 3 mL of heptane were added, the mixture was vortexed and then centrifuged at 1000× *g* for 10 min. The upper heptane layer was transferred into tubes containing anhydrous Na_2_SO_4_ to remove traces of water. The top layer was finally collected in 2 mL GC vials and stored at −20 °C until GC analysis.

The procedure was done in triplicate for each sample.

The free fatty acid content in the sample was calculated from the difference between the two results (TFA and EFA) on the same sample.

##### GC Analysis of Fatty Acid Methyl Esters (FAMEs)

The fatty acid compositions of the samples were determined using a gas chromatograph system (GC-2010 Plus, Shimadzu, Kyoto, Japan) equipped with a flame ionization detector and an AOC-20I auto-injector. The column used was a 60 m RTX^®^-2330 GC column (Restek, Bellefonte, USA; 0.25 mm internal diameter with a 0.10 µm film thickness). The injection volume was 1 µL and the carrier gas was hydrogen gas at a linear velocity of 40 cm/s. The split ratio was 50:1. The initial oven temperature profile was 125 °C for 3 min, was increased to 220 °C at the rate of 2 °C/min and then maintained for 5 min. The temperatures of the injector and the detector were 260 °C and 265 °C, respectively. The peaks of individual fatty acids were identified and quantified based on the retention time of an internal standard (C23:0) (T9900, Sigma Aldrich, Saint Louis, MO, USA), an external standard mixture (Supelco FAME mix C4-C24) (Sigma Aldrich, Saint Louis, MO, USA), and theoretical flame ionization detector response factors. Relevant algorithms to quantify the FAMEs were standard (AOCS Ce 1f-96, Ce 1h-05 and Ce 1i-07). Unresolved fatty acids were not reported.

##### Calculation of Free Fatty Acids (FFA)

The amounts of individual free fatty acids (mg fatty acid/g cooked meat) were determined in the digests after 180 min of in vitro gastro-small intestinal digestion as described by Zhu et al. [26]:

Individual FFA after 180 min of simulated digestion (mg/g cooked meat) = Individual total fatty acid (TFA) at 180 min – individual esterified EFA at 180 min.

##### Desaturase Indices and *n*−6/*n*−3 Fatty Acid Ratios

The stearoyl-CoA desaturase index has been reported as the product/precursor ratio of FA as described by Alarcón et al. [29]:

DI_16_ = C16:1/C16:0

and

DI_18_ = C18:1 − cis9/C18:0

The *n*−6/*n*−3 FA ratio was calculated as:

Linoleic acid (LA) + arachidonic acid (ARA)/linolenic acid (ALA) + eicosapentaenoic acid (EPA) + docosapentaenoic acid (DPA) + docosahexaenoic acid (DHA).

#### 2.2.6. Statistical Analysis

All the experiments were carried out in triplicate for 2 types of meat cuts, each from *n* = 5 pasture-raised and *n* = 5 grain-finished animals. Statistical evaluation was performed using a general linear model. The average of three replicates for each animal were taken for performing a two-way analysis of variance (ANOVA) using OriginPro software (OriginLab Corporation, Northampton, MA, USA), with a Tukey’s test for estimating the significance of differences among the production systems and meat cuts (*p* < 0.05). Results obtained from the statistical analysis are reported as the means and the standard error of the means.

## 3. Results and Discussion

### 3.1. Physicochemical Analysis of Meat

#### 3.1.1. Cook Loss

Cook loss is the phenomenon that causes the meat to lose volume and weight through the process of fluid exudation during the cooking process. Cook loss measures the ability of a food matrix to bind water and fat after the denaturation and aggregation of protein molecules. This change in fluid content, along with modifications of texture-forming properties of the proteins and fats in meat, leads to a variation in meat quality attributes [30].

The overall cook loss values for the meat cuts showed similar trends to those reported by Purchas and Wilkinson [17] (Table 1). There were no significant differences (*p* > 0.05) in cook loss for meat from the different production systems (pasture-raised and grain-finished). Striploin had lower cook loss than tenderloin, but the difference was only significant (*p* < 0.05) for the pasture-raised group. Schönfeldt and Strydom [31] have also reported differences in cook loss between meat cuts, which have been related to the variations in sample dimensions and composition along with the spatial distribution of fat or lean meat areas, and the meat surface properties. These may affect how meat proteins or fat behave when exposed to heat, such as protein denaturation, shrinkage, and fat-melting [32]. Importantly, striploin was cooked with the subcutaneous fat layer on (to reflect normal culinary procedure), which could partly explain the observed differences in cook loss between the two meat cuts.

#### 3.1.2. Moisture Content and pH

The water in meat is mostly bound to protein molecules and is usually found between and within muscle cells and muscle bundles. As the temperature increases the tertiary and secondary structures unfold, lose or rearrange their disulfide bridges, undergo modifications in their side chains, cross-link with other polypeptides, and lose the surrounding water [30].

The moisture content of the cooked meat depends on factors such as the type of meat cut, cooking temperature, final internal temperature, and portion size. The moisture content remaining in a product after cooking has been reported to be one of the major contributors to the sensation of juiciness [31]. The moisture contents of cooked meat samples were generally linked to the cooking loss, in the present study. Pasture-raised cooked tenderloin samples had more cook loss and significantly (*p* < 0.05) lower moisture content than pasture-raised striploin samples (Table 1) while no differences in cook loss or moisture content could be seen among the two meat cuts from grain-finished animals. It is also important to mention that despite having no significant differences in cook loss among pasture-raised and grain-finished meat samples, both striploin and tenderloin samples from pasture-raised animals had higher moisture content than their respective grain-finished counterparts. The higher moisture content of pasture-raised meat could partly be explained by their lower fat contents than grain-finished meat (discussed in Section 3.1.4).

No significant effect (*p* > 0.05) of the meat production system could be observed on the cooked meat pH (Table 1) whereas the meat cut type significantly (*p* < 0.05) influenced the meat pH, possibly due to differences in rates of metabolism of the different muscles post-mortem.

#### 3.1.3. Protein Content

Slight but significant (*p* < 0.05) differences in the total crude protein were observed for striploin samples from the two production systems (Table 1). No such significant (*p* > 0.05) differences could, however, be observed among the tenderloin samples. These results were also confirmed by the observed significant interactions (*p* < 0.05) between meat cut type and the production system. Among the meat cuts, tenderloin from grain-finished animals had significantly (*p* < 0.05) lower protein content than the corresponding striploin samples while the pasture-raised tenderloin and striploin had similar protein contents, which could be explained by the higher fat content of tenderloin samples from the grain-finished animal group. The values of protein content reported in this study are similar to those noted by Purchas and Wilkinson [17] for New Zealand striploin and tenderloin meat cuts.

#### 3.1.4. Fat Content

The meat cuts used in this study were chosen based on their relatively low fat content. Several studies have shown differences in fat content among retail beef cuts, while in red meat the loin is regarded as the leanest portion of the carcass [33]. Cuts like striploin have been reported to have varying fat content, which could be due to the inclusion of the subcutaneous fat layer [34]. However, the subcutaneous fat layer was removed after cooking in this study to follow the common eating practice, thereby reducing the fat content, which could be the reason for not finding any significant differences (*p* > 0.05) among the striploin and tenderloin meat samples within each production system. However, an overall significant effect (*p* < 0.05) of the meat cut type was observed on the fat content of the cooked meat. As expected, the meat cuts from the grain-finished animals had a much higher fat content (*p* < 0.05) than those from the pasture-raised animals (Table 1). These results agree with long-known facts on the influence of grazing systems on meat fat. Pasture feeding has been reported to lead to a leaner carcass, reduce intramuscular fat deposition, and improve the fatty acid profile of beef lipids [12].

### 3.2. Protein Digestibility

#### 3.2.1. Ninhydrin-Reactive Free Amino Nitrogen

The proteins of the food matrix were broken down during digestion into smaller peptides. The rate of protein hydrolysis is represented by the amount of reactive amino nitrogen released during gastro-small intestinal in vitro digestion (Table 2). During the gastric digestion phase, there was a minimal increase in free amino groups. Pepsin cleaves protein molecules into smaller peptides and is generally responsible for about 15% of protein hydrolysis during gastro-small intestinal digestion [35].

The acidic pH of the simulated gastric fluid may have induced the formation of gastric chyme with coagulated meat proteins that were resistant to further protein hydrolysis by pepsin [36]. However, within the first 10 min of the small intestinal digestion, a steep rise in free amino groups was observed. This could be due to greater protein solubilization in a neutral pH environment [36]. Moreover, the pancreatic enzymes present during small intestinal digestion contain peptidases such as trypsin, chymotrypsin, and carboxypeptidase, which cleave the larger polypeptides produced by pepsin hydrolysis into smaller peptides. The wider specificity of the pancreatic proteases for peptide bonds makes small intestinal digestion more efficient, resulting in products with only 6–8 amino acids on average.

No significant (*p* > 0.05) effects of the animal feeding system for any of the meat cuts on the overall release of free amino N could be seen during digestion after simulated digestion for 180 min. However, tenderloin and striploin samples from pasture-raised animals had slightly lower but statistically significant (*p* < 0.05; Table 2) ninhydrin-reactive N values after 60 min of digestion than those from grain-finished animals. Between the two meat cuts, tenderloin showed significantly (*p* < 0.05) higher protein hydrolysis than striploin throughout digestion (Table 2).

#### 3.2.2. Tricine SDS-PAGE

**Figure 1:**
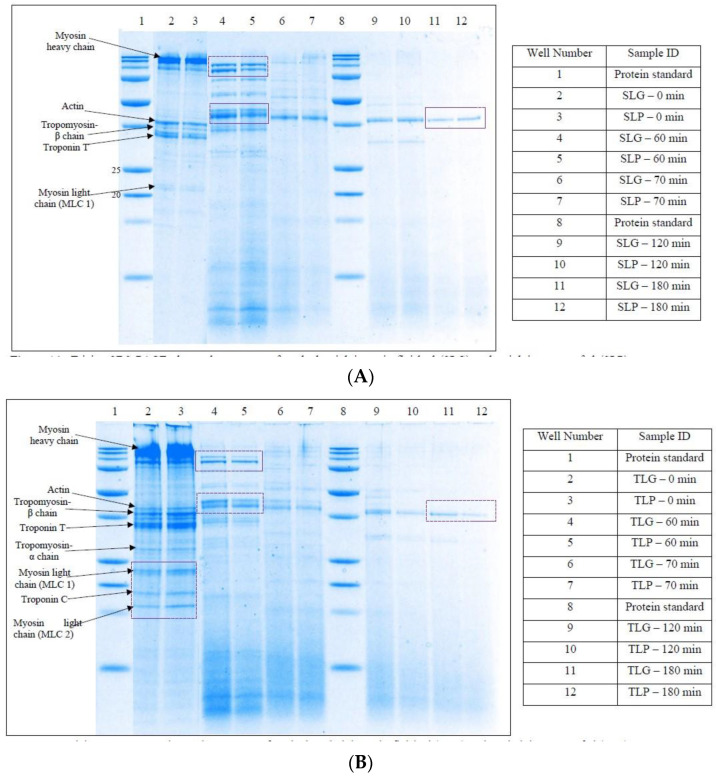
(**A**) Tricine SDS-PAGE electrophoretogram of cooked striploin grain-finished (SLG) and striploin pasture-fed (SLP) meat digests after 0, and 60 min of simulated oral-gastric digestion and 70, 120, and 180 min of simulated gastro-small intestinal digestion following initial 2 min of oral digestion. Bands in the molecular weight protein standard lane correspond to molecular weights 250, 150, 100, 75, 50, 37, 25, 20, 15, and 10 kDa. (**B**) Tricine SDS-PAGE electrophoretogram of cooked tenderloin grain-finished (TLG) and tenderloin pasture-fed (TLP) meat digests after 0, and 60 min of simulated oral-gastric digestion and 70, 120, and 180 min of simulated gastro-small intestinal digestion following initial 2 min of oral digestion. Bands in the molecular weight protein standard lane correspond to molecular weights 250, 150, 100, 75, 50, 37, 25, 20, 15, and 10 kDa.

The digestion of soluble proteins and peptides that have a molecular weight > 1 kDa was determined through reduced tricine SDS-PAGE. Figure 1A,B provides information regarding the breakdown of meat and meat alternative proteins by digestive enzymes. The digests from both the meat cuts for pasture-raised and grain-finished meat did not show any noticeable differences in protein breakdown profiles and peptide release patterns. The higher molecular weight (HMW) proteins which correspond to myosin heavy chain (220 kDa) observed at 0 min of gastric digestion were observed to be digested during 60 min of gastric digestion in both striploin and tenderloin. Some of the other meat proteins such as actin (43 kDa), tropomyosin (39 kDa), troponin (35 kDa), and myosin light chain (23 kDa) were identified at 0 min of gastric digestion (Kaur et al., 2014). Small peptides with low molecular weight (<25 kDa) were also formed during 60 min of gastric digestion.

Small intestinal digestion was marked by a rapid decrease in band intensity of large meat proteins with molecular weight <100 kDa. By 180 min of gastro-small intestinal digestion, most of the proteins and peptides were digested except for a few bands as shown in Figure 1A,B. Similar results for digested beef muscle were observed by Kaur et al. [36]. A greater reduction in intensities of some bands (marked in Figure 1B) was observed for the pasture-raised tenderloin digests when compared to grain-finished tenderloin digests after 60, 120, and 180 min, showing faster protein breakdown. Among tenderloin and striploin, tenderloin appeared to show greater protein breakdown during digestion, which agrees with the two-way ANOVA results that showed significant differences (*p* < 0.05) between the free amino N% of tenderloin and striploin meat cuts after 0, 60, and 180 min of gastro-small intestinal digestion (Table 2).

### 3.3. Fatty Acid Profiles of Control Meat Samples and Their Digests

The meat samples used in this study were trimmed of all extra-muscular fat before in vitro digestion; thus, the only source of fat was the intramuscular fat. Several studies indicate pasture-raised beef to be leaner in comparison to grain-finished beef in terms of intramuscular fat composition [37,38]. The data presented in Table 1 confirm these reports and shows significantly higher (*p* < 0.05) total fat contents for grain-finished meat cuts.

#### 3.3.1. Total Fatty Acid (TFA) Profiles of Cooked Meat

Fatty acid compositions differed among the cooked meat samples from the two production systems (Table 3). The total fatty acid (TFA) content for oleic acid was observed to be the highest in undigested cooked meat followed by palmitic and stearic acids, for both pasture-raised and grain-finished animals. The concentrations of individual saturated total fatty acids (SFAs) and individual monounsaturated total fatty acids (MUFAs) and *n*−6 PUFAs were generally higher in the meat from grain-finished animals. No significant differences (*p* > 0.05) could be observed in the individual SFAs or MUFAs (as shown in Table 3) among tenderloin and striploin, except for stearic acid.

The *n*−3 PUFAs, particularly EPA, ALA, and DPA, were present in greater amounts in cooked meat from pasture-raised than in grain-finished beef. Tenderloin samples showed significantly (*p* < 0.05) higher individual *n*−6 and *n*−3 PUFAs than striploin. Meat from animals that are fed grain-based diets have been reported to contain higher concentrations of *n*−6 PUFAs while pasture-raised animals have greater amounts of *n*−3 PUFAs [6,9]. The differences in fat composition of the digests observed in this study are broadly in line with the observations of Clancy [39] for pasture-raised beef and milk samples.

#### 3.3.2. Free Fatty Acid (FFA) Profiles, Ratios and Desaturase Indices of Cooked Meat Digests

The individual free fatty acid profiles of meat digests after 180 min of digestion are shown in Table 4 and Table 5. It is clear from the amounts of free fatty acids released during digestion, in comparison to the total amounts of individual fatty acids present in the undigested meat samples (Table 3), that the digestion of fatty acids was not complete by the end of the small intestinal digestion phase that consisted of one hour of gastric and two hours of small-intestinal digestion. In humans, the normal small-intestinal transit time is 3–4 h [40], which is longer than the duration employed in the present study. The in vitro digestion protocol used in the current study was based on previously published established static digestion protocols [23,24] and included two hours of simulated small intestinal digestion. This is a compromise between the longer time of digestion and the fact that the model becomes less meaningful over time because the digestion products are not being removed, as would be the case in vivo.

The total amount of free oleic acid released per gram of meat was the highest for both tenderloin and striploin digests followed by palmitic and stearic acids. This agrees with a study by Smith and Johnson [41]. Digested meat samples from grain-finished animals had significantly (*p* < 0.05) higher free palmitic acid content than those from pasture-raised animals. The amounts of almost all the individual free MUFAs released after digestion were significantly (*p* < 0.05) higher for grain-finished meats than for their pasture-raised meat counterparts (Table 4 and Table 5). As expected, the amounts of *n*−3 PUFAs, namely free ALA, EPA, and DPA were mostly higher in the digests from pasture-raised animals.

The role of SFAs in increasing the risk of many conditions such as obesity and cardiovascular disease and the role of long-chain *n*−3 PUFAs in providing health benefits has been reported in the literature, although the former is still under scrutiny [42]. The results in Table 4 and Table 5 point toward the advantages of consuming pasture-raised meat over grain-finished meat, as pasture-raised meat provides higher amounts of free long-chain (LC) *n*−3 PUFAs (particularly EPA and DPA) after 180 min of digestion.

Among the two meat cuts, no significant differences (*p* > 0.05) could be observed for the amounts of individual free LC*n*−3 PUFAs released during digestion. However, tenderloin samples in general released higher amounts of most of the individual SFAs, MUFAs, and *n*−6 PUFAs along with ALA during digestion than the striploin samples (Table 4) despite showing no significant differences among the total fat contents and the saturated (except for stearic acid) and mono-unsaturated fatty acid profiles among the respective control undigested meat samples (Table 1 and Table 3). This agrees with the higher rates of free amino N release and higher protein breakdown for tenderloin during digestion, showing that the higher rates of digestion of protein may influence the release of fats from within the structures and thus enhance their digestion and bioaccessibility.

The free fatty acid ratios and desaturase indices presented in Table 6 serve as health indication markers [29,43]. Our results showed a higher ratio (≥4-fold) for digested grain-finished tenderloin or striploin while the respective digested pasture-raised meat counterparts reported significantly (*p* < 0.05) lower *n*−6/*n*−3 ratios. Diets with low *n*−6/*n*−3 ratios (4:1) have been associated with better neurogenesis, reduced depression, and other cognitive benefits [42,43,44].

The delta-9 desaturase enzymes (also known as stearoyl-CoA desaturases, SCD) play an important role in lipid metabolism by catalyzing the conversion of saturated (SFA) to monounsaturated (MUFA) fatty acids by introducing a cis double bond at the delta-9 position [45]. Saturated fatty acids such as myristic, palmitic, and stearic acids are mainly used as substrates which are converted into myristoleic, palmitoleic, and oleic acids, respectively. Oleic and palmitoleic acids are the major MUFAs in fat depots and membrane phospholipids. Very high activities of estimated SCD-16 (as the ratio of palmitoleic to palmitic acid) and SCD-18 (as the ratio of oleic to stearic acid) have been linked with obesity and other metabolic disorders [29,45,46,47]. Both desaturase indices, DI16 and DI18, were found to be significantly (*p* < 0.05) lower for pasture-raised meat digests, particularly for tenderloin than for grain-finished meat digests.

## 4. Conclusions

The objectives of this study were to determine and compare the nutritional value, protein digestibility, and free fatty acid release during the digestion of tenderloin and striploin from grain-finished and pasture-raised beef by utilizing an in vitro digestion model. When comparing the meat cuts, tenderloin showed slightly but significantly higher (*p* < 0.05) overall protein hydrolysis than striploin, in terms of free amino N release during digestion. However, no significant effect of the animal feeding system was observed on the overall protein hydrolysis of meat during simulated digestion, irrespective of the meat cut. SDS-PAGE results showed that tenderloin from pasture-raised samples had higher and faster protein breakdown during digestion than striploin from pasture-raised animals.

The amounts of individual free fatty acids in the digests of the meats from pasture-raised and grain-finished production systems differed and were largely reflective of the composition of the triglycerides that were being digested. The total amounts of free SFAs and MUFAs were higher in the grain-finished meat samples. This suggests that pasture-raised meat was likely to have lower risks of contributing to chronic diseases, such as cardiovascular disease, which are related to levels of SFAs (lauric, myristic, and palmitic acids). The pasture-raised meat digests from both striploin and tenderloin contained slightly but significantly (*p* < 0.05) higher amounts of free long-chain *n*−3 fatty acids (particularly EPA and DPA) which have been extensively studied for their beneficial health effects. Tenderloin samples in general released higher amounts of most of the individual free SFAs, MUFAs, and *n*−6 PUFAs during digestion than the striploin samples. This agrees with the higher rates of free amino N release and higher protein breakdown for tenderloin during digestion, showing that the higher rates of digestion of protein may influence the release of fats from within the structures and thus enhance their digestion and bioaccessibility.

For each meat cut type, significantly lower *n*−6/*n*−3 PUFA ratios (*p* < 0.05) and desaturase indices (*p* < 0.001) were observed for digests from pasture-raised than the counterpart digests from grain-finished animals. The overall effect of the production system on the *n*−6/*n*−3 ratio or desaturase indices (except for DI18) for meat digests was, however, not significant (*p* > 0.05). For *n*−6/*n*−3 PUFA ratio and desaturase indices, significant interactions (*p* < 0.001) between the meat cut type and the production system were observed, indicating that the differences observed among the production systems were not consistent for each meat cut. For striploin digests, the differences in the mean values for these parameters between the two production systems were slightly larger than those for tenderloin digests, suggesting that striploin was more influenced by the meat production system than tenderloin. The values for the desaturase indices and *n*−6/*n*−3 ratios for tenderloin digests from the pasture-raised animals were the lowest among all the samples, suggesting better health benefits when consuming pasture-raised meat, particularly tenderloin. The findings of this study are currently being confirmed through a long-term clinical study.

## Figures and Tables

**Table 1:** Physicochemical analysis of cooked striploin (SL) and tenderloin (TL) from pasture-raised and grain-finished production systems.

Parameters	Muscle Type	Production System		SEM	Meat Cut	Production System	Interaction
		Pasture-Raised	Grain-Finished				
Cook loss (%)	SL	14.58 ^aY^	15.74 ^aX^	0.50	*p* < 0.001	*p* > 0.05	*p* > 0.05
	TL	18.67 ^aX^	18.00 ^aX^	0.49			
Moisture content (%)	SL	66.67 ^aX^	63.49 ^bX^	0.01	*p* < 0.05	*p* < 0.05	*p* > 0.05
	TL	64.14 ^aY^	61.47 ^bX^	0.01			
Protein content (%)	SL	28.12 ^bX^	29.28 ^aX^	0.31	*p* < 0.05	*p* > 0.05	*p* < 0.05
	TL	28.39 ^aX^	27.13 ^aY^	0.43			
Fat content (%)	SL	4.97 ^bX^	7.70 ^aX^	0.64	*p* < 0.05	*p* < 0.001	*p* > 0.05
	TL	6.43 ^bX^	10.30 ^aX^	0.87			
pH	SL	5.97 ^aX^	5.94 ^aX^	0.03	*p* < 0.05	*p* > 0.05	*p* > 0.05
	TL	5.87 ^aX^	5.85 ^aX^	0.02			

Production system—Pasture-raised, *n* = 5; Grain-finished, *n* = 5. SEM—Standard error (Pooled). ^ab^ Means within a row with the same superscript letter are not different (*p* > 0.05) between production systems. ^XY^ Means within each parameter column with the same superscript letter are not different between muscle types (*p* > 0.05).

**Table 2:** Free amino N (%) release during simulated gastro-small intestinal digestion of pasture-fed and grain-finished cooked striploin and tenderloin.

Free Amino Nitrogen (%)	Muscle Type	Production System		SEM	Meat Cut	Production System	Interaction
		Pasture-Raised	Grain-Finished				
0 min	SL	1.65 ^aX^	1.66 ^aX^	0.06	*p* < 0.05	*p* > 0.05	*p* > 0.05
	TL	2.22 ^aY^	2.09 ^aY^	0.14			
60 min	SL	4.24 ^aX^	4.42 ^bX^	0.12	*p* < 0.001	*p* < 0.05	*p* > 0.05
	TL	4.53 ^aY^	5.42 ^bY^	0.16			
180 min	SL	13.06 ^aX^	12.88 ^aX^	0.29	*p* < 0.05	*p* > 0.05	*p* > 0.05
	TL	13.55 ^aY^	14.79 ^aY^	0.34			

Digestion times 0 and 60 are the gastric digestion times following 2 min of oral digestion. 180 min is the total digestion time in gastro-small intestinal digestion following 2 min of oral digestion. Production system—Pasture-raised, *n* = 5; Grain-finished, *n* = 5. SEM—Standard error (Pooled). ^ab^ Means within a row with the same superscript letter are not different (*p* > 0.05) between production systems. ^XY^ Means within each parameter column with the same superscript letter are not different between muscle types (*p* > 0.05).

**Table 3:** Total fatty acid (TFA, selected) contents for cooked striploin (SL) and tenderloin (TL) from grain-finished and pasture-raised animals.

Fatty Acids		Muscle Type	Total Fatty Acid (mg/g Cooked Meat)		SEM	Meat Cut	Production System	Interaction
			Pasture-Raised	Grain-Finished				
*Individual SFA*								
C14:0	Myristic acid	SL	1.08 ^bX^	1.68 ^aX^	0.13	*p* > 0.05	*p* < 0.05	*p* > 0.05
		TL	1.08 ^bX^	1.85 ^aX^	0.20			
C16:0	Palmitic acid	SL	13.69 ^bX^	15.09 ^aX^	0.62	*p* > 0.05	*p* < 0.05	*p* > 0.05
		TL	13.56 ^bX^	19.53 ^aX^	1.65			
C17:0	Heptadecanoic acid	SL	0.57 ^bX^	0.99 ^aX^	0.09	*p* > 0.05	*p* < 0.001	*p* > 0.05
		TL	0.70 ^bX^	1.29 ^aX^	0.13			
C18:0	Stearic acid	SL	10.05 ^aX^	8.10 ^aX^	0.48	*p* < 0.05	*p* > 0.05	*p* > 0.05
		TL	12.49 ^aY^	12.75 ^aY^	0.87			
*Individual MUFA*								
C14:1	Myristoleic acid	SL	0.17 ^bX^	0.42 ^aX^	0.05	*p* > 0.05	*p* < 0.001	*p* > 0.05
		TL	0.15 ^bX^	0.38 ^aX^	0.05			
C16:1	Palmitoleic acid	SL	1.20 ^bX^	1.95 ^aX^	0.15	*p* > 0.05	*p* < 0.001	*p* > 0.05
		TL	0.99 ^bX^	2.08 ^aX^	0.23			
C18:1 *c*9	Oleic acid	SL	19.32 ^bX^	28.57 ^aX^	1.75	*p* > 0.05	*p* < 0.001	*p* > 0.05
		TL	18.34 ^bX^	32.89 ^aX^	3.26			
C18:1 *c*11	*cis*-Vaccenic acid	SL	1.70 ^bX^	0.72 ^aX^	0.19	*p* > 0.05	*p* < 0.001	*p* > 0.05
		TL	1.39 ^bX^	0.58 ^aX^	0.14			
*Individual PUFA*								
C18:2 *n*−6	Linoleic acid	SL	0.88 ^bY^	1.49 ^aY^	0.11	*p* < 0.001	*p* < 0.001	*p* > 0.05
		TL	1.65 ^bX^	2.46 ^aX^	0.18			
C18:2 *c*9, *t*11	Conjugated linoleic acid	SL	0.25 ^aY^	0.13 ^aY^	0.02	*p* < 0.05	*p* > 0.05	*p* > 0.05
		TL	0.26 ^aX^	0.31 ^aX^	0.04			
C18:3 *n*−3	α-Linolenic acid	SL	0.52 ^bY^	0.23 ^aY^	0.06	*p* < 0.001	*p* < 0.001	*p* > 0.05
		TL	0.91 ^bX^	0.45 ^aX^	0.09			
C20:5 *n*−3	Eicosapentaenoic acid (EPA)	SL	0.20 ^bY^	0.15 ^aY^	0.01	*p* < 0.001	*p* < 0.001	*p* > 0.05
		TL	0.31 ^bX^	0.20 ^aX^	0.02			
C22:5 *n*−3	Docosapentaenoic acid (DPA)	SL	0.31 ^bY^	0.27 ^aY^	0.01	*p* < 0.001	*p* < 0.001	*p* > 0.05
		TL	0.47 ^bX^	0.34 ^aX^	0.02			
C22:6 *n*−3	Docosahexaenoic acid (DHA)	SL	0.03 ^aX^	0.03 ^aX^	0.00	*p* < 0.001	*p* > 0.05	*p* > 0.05
		TL	0.05 ^aY^	0.05 ^aY^	0.00			

SFA: saturated fatty acids; MUFA: monounsaturated fatty acids; *c*: *cis*; *t*: *trans*; PUFA: polyunsaturated fatty acids; Pasture-raised, *n* = 5; Grain-finished, *n* = 5. SEM—Standard error (Pooled). ^ab^ Means within a row with the same superscript letter are not different (*p* > 0.05) between production systems. ^XY^ Means within each parameter column with the same superscript letter are not different between muscle types (*p* > 0.05).

**Table 4:** Individual free fatty acids (selected) released after 180 min of simulated gastro-small intestinal digestion for cooked striploin (SL) and tenderloin (TL) from grain-finished and pasture-raised animals.

Fatty Acids		Muscle Type	Free Fatty Acid Released (mg/g Cooked Meat)		SEM	Meat Cut	Production System	Interaction
			Pasture-Raised	Grain-Finished				
*Individual SFA*								
C14:0	Myristic acid	SL	0.22 ^bY^	0.47 ^aX^	0.04	*p* < 0.05	*p* < 0.05	*p* > 0.05
		TL	0.41 ^bX^	0.90 ^aX^	0.13			
C16:0	Palmitic acid	SL	3.75 ^bY^	5.48 ^aY^	0.34	*p* < 0.05	*p* < 0.05	*p* > 0.05
		TL	5.61 ^bX^	10.47 ^aX^	1.20			
C17:0	Heptadecanoic acid	SL	0.14 ^bY^	0.33 ^aY^	0.04	*p* < 0.05	*p* < 0.001	*p* > 0.05
		TL	0.27 ^bX^	0.67 ^aX^	0.09			
C18:0	Stearic acid	SL	2.33 ^aY^	2.50 ^aY^	0.17	*p* < 0.001	*p* < 0.05	*p* > 0.05
		TL	4.43 ^aX^	6.35 ^aX^	0.62			
*Individual MUFA*								
C14:1	Myristoleic acid	SL	0.05 ^bX^	0.13 ^aX^	0.01	*p* > 0.05	*p* < 0.05	*p* > 0.05
		TL	0.06 ^bX^	0.15 ^aX^	0.03			
C16:1	Palmitoleic acid	SL	0.31 ^bX^	0.70 ^aX^	0.07	*p* > 0.05	*p* < 0.05	*p* > 0.05
		TL	0.41 ^bX^	1.03 ^aX^	0.15			
C18:1 *c*9	Oleic acid	SL	4.88 ^bY^	8.92 ^aX^	0.78	*p* < 0.05	*p* < 0.05	*p* > 0.05
		TL	7.32 ^bX^	16.76 ^aX^	2.39			
C18:1 *c*11	*cis*-Vaccenic acid	SL	0.18 ^bY^	0.45 ^aY^	0.05	*p* < 0.05	*p* < 0.001	*p* > 0.05
		TL	0.29 ^bX^	0.84 ^aX^	0.12			
*Individual PUFA*								
C18:2 *n*−6	Linoleic acid	SL	0.56 ^bY^	0.68 ^aY^	0.03	*p* < 0.001	*p* < 0.05	*p* > 0.05
		TL	0.76 ^bX^	1.14 ^aX^	0.08			
C18:2 *c*9, *t*11	Conjugated linoleic acid	SL	0.05 ^aY^	0.05 ^aX^	0.01	*p* < 0.05	*p* > 0.05	*p* > 0.05
		TL	0.08 ^aX^	0.12 ^aX^	0.02			
C18:3 *n*−3	α-Linolenic acid	SL	0.13 ^aY^	0.05 ^bY^	0.01	*p* < 0.001	*p* < 0.001	*p* > 0.05
		TL	0.28 ^aX^	0.19 ^bX^	0.03			
C20:5 *n*−3	Eicosapentaenoic acid (EPA)	SL	0.05 ^aX^	0.03 ^bX^	0.00	*p* < 0.05	*p* > 0.05	*p* > 0.05
		TL	0.08 ^aX^	0.05 ^bX^	0.01			
C22:5 *n*−3	Docosapentaenoic acid (DPA)	SL	0.07 ^aX^	0.06 ^aX^	0.00	*p* < 0.05	*p* < 0.05	*p* > 0.05
		TL	0.10 ^aX^	0.06 ^bX^	0.01			
C22:6 *n*−3	Docosahexaenoic acid (DHA)	SL	0.02 ^aX^	0.01 ^aX^	0.00	*p* < 0.05	*p* > 0.05	*p* > 0.05
		TL	0.02 ^aX^	0.02 ^aX^	0.00			

SFA: saturated fatty acids; MUFA: monounsaturated fatty acids; *c*: *cis*; *t*: *trans*; PUFA: polyunsaturated fatty acids. Pasture-raised, *n* = 5; Grain-finished, *n* = 5. SEM—Standard error (Pooled). ^ab^ Means within a row with the same superscript letter are not different (*p* > 0.05) between production systems. ^XY^ Means within each parameter column with the same superscript letter are not different between muscle types (*p* > 0.05).

**Table 5:** Sum of the released free saturated (SFA), mono- (MUFA) and polyunsaturated fatty acid (PUFA) amounts for cooked striploin (SL) and tenderloin (TL) digests from pasture-raised and grain-finished animals after 180 min of digestion under simulated gastro-small intestinal conditions.

Free Fatty Acids	Muscle Type	Free Fatty Acid Released (mg/g Cooked Meat)		SEM	Meat Cut	Production System	Interaction
		Pasture-Raised	Grain-Finished				
Σ SFA	SL	6.50 ^bY^	8.89 ^aY^	0.53	*p* < 0.001	*p* > 0.05	*p* < 0.05
	TL	10.87 ^bX^	18.65 ^aX^	2.05			
Σ MUFA	SL	5.49 ^bY^	10.51 ^aX^	0.94	*p* < 0.05	*p* > 0.05	*p* < 0.05
	TL	8.19 ^bX^	19.27 ^aX^	2.76			
Σ PUFA	SL	1.09 ^aY^	1.08 ^aY^	0.04	*p* < 0.001	*p* > 0.05	*p* > 0.05
	TL	1.54 ^aX^	1.80 ^aX^	0.11			
Σ *n*−6 PUFA	SL	0.78 ^aY^	0.87 ^aY^	0.03	*p* < 0.001	*p* > 0.05	*p* > 0.05
	TL	0.98 ^bX^	1.35 ^aX^	0.09			
Σ *n*−3 PUFA	SL	0.27 ^aY^	0.15 ^bY^	0.02	*p* < 0.001	*p* > 0.05	*p* < 0.001
	TL	0.47 ^aX^	0.32 ^bX^	0.04			
Σ LC*n*−3 PUFA	SL	0.14 ^aX^	0.10 ^bX^	0.01	*p* < 0.05	*p* < 0.05	*p* > 0.05
	TL	0.19 ^aX^	0.13 ^bX^	0.02			
Σ EPA, DHA	SL	0.06 ^aX^	0.04 ^bX^	0.00	*p* < 0.05	*p* > 0.05	*p* > 0.05
	TL	0.09 ^aX^	0.07 ^bX^	0.01			

SFA: saturated fatty acids; MUFA: monounsaturated fatty acids; PUFA: polyunsaturated fatty acids; EPA: eicosapentaenoic acid; DHA: docosahexaenoic acid; LC *n*−3 PFA: EPA+DHA+docosapentaenoic acid. Pasture-raised, *n* = 5; Grain-finished, *n* = 5. SEM—Standard error (Pooled). ^ab^ Means within a row with the same superscript letter are not different (*p* > 0.05) between production systems. ^XY^ Means within each parameter column with the same superscript letter are not different between muscle types (*p* > 0.05).

**Table 6:** Fatty acid ratios and desaturase indices (DI) for cooked striploin (SL) and tenderloin (TL) digests from pasture-raised and grain-finished animals after 180 min of digestion under simulated gastro-small intestinal conditions.

Free Fatty Acids	Muscle Type	Free Fatty Acid Released (mg/g Cooked Meat)		SEM	Meat Cut	Production System	Interaction
		Pasture-Raised	Grain-Finished				
*FA ratios and DI*							
*n*−6/*n*−3 PUFA	SL	2.88 ^bX^	5.69 ^aX^	0.48	*p* < 0.05	*p* > 0.05	*p* < 0.001
	TL	2.11 ^bY^	4.24 ^aY^	0.36			
DI_16_	SL	0.08 ^bX^	0.13 ^aX^	0.01	*p* < 0.001	*p* > 0.05	*p* < 0.001
	TL	0.07 ^bX^	0.10 ^aY^	0.01			
DI_18_	SL	2.14 ^bX^	3.63 ^aX^	0.29	*p* < 0.001	*p* < 0.05	*p* < 0.001
	TL	1.66 ^bY^	2.56 ^aY^	0.18			

FA: fatty acid; PUFA: polyunsaturated fatty acids; Pasture-raised, *n* = 5; Grain-finished, *n* = 5. SEM—Standard error (Pooled). ^ab^ Means within a row with the same superscript letter are not different (*p* > 0.05) between production systems. ^XY^ Means within each parameter column with the same superscript letter are not different between muscle types (*p* > 0.05). DI_16_ and DI_18_ were calculated as described in Section Desaturase Indices and *n*−6/*n*−3 Fatty Acid Ratios.

## Data Availability

The datasets generated for this study are available on request to the corresponding author.

## Supplementary Materials

The following supporting information can be downloaded at: https://www.mdpi.com/article/10.3390/foods12061239/s1, Table S1: Details of carcasses used for this study.
