# Supplementary material for: Protein and Lipid Digestibility of Pasture-Raised and Grain-Finished Beef: An In Vitro Comparison
Source: Foods. 2023 Mar 14;12(6):1239. doi: 10.3390/foods12061239 (PMC10047994; doi:10.3390/foods12061239)
Supplement: Supplementary file 1 [file foods-12-01239-s001.zip › foods-2091962-supplementary.pdf]

Table S1. Detailsofcarcassesusedforthestudy.

**Productionsystem**	**Type**	**CarcassLiveWeight(kg)**	**Gradedweight(kg)**	**pH**
Pasture	Steer	618	343	5.57
Pasture	Steer	615	342	5.55
Pasture	Steer	611	341	5.55
Pasture	Steer	609	343	5.80
Pasture	Steer	606	335	5.57
Grain	Steer	616	326	5.47
Grain	Steer	612	333	5.45
Grain	Steer	604	337	5.45
Grain	Steer	612	340	5.46
Grain	Steer	598	324	5.46
